# Proapoptotic *Bad* Involved in Brain Development, When Severely Defected, Induces Dramatic Malformation in Zebrafish

**DOI:** 10.3390/ijms22094832

**Published:** 2021-05-02

**Authors:** Jo-Chi Hung, Jen-Leih Wu, Jiann-Ruey Hong

**Affiliations:** 1 Laboratory of Molecular Virology and Biotechnology, Institute of Biotechnology, National Cheng Kung University, Tainan 701, Taiwan; betty01269@gmail.com; 2 Department of Biotechnology and Bioindustry Sciences, National Cheng Kung University, Tainan 701, Taiwan; 3 Laboratory of Marine Molecular Biology and Biotechnology, Institute of Cellular and Organismic Biology, Academia Sinica, Nankang, Taipei 115, Taiwan; jlwu@gate.sinica.edu.tw

**Keywords:** bad, brain defect, environmental stress, p53/caspase-8 death signaling, gene knockdown

## Abstract

The BH3-only molecule Bad regulates cell death via its differential protein phosphorylation, but very few studies address its effect on early embryonic development in vertebrate systems. In this work, we examined the novel role of zebrafish Bad in the initial programmed cell death (PCD) for brain morphogenesis through reducing environmental stress and cell death signaling. Bad was considered to be a material factor that because of the knockdown of Bad by morpholino oligonucleotides, PCD was increased and the reactive oxygen species (ROS) level was enhanced, which correlated to trigger a p53/caspase-8 involving cell death signaling. This Bad knockdown-mediated environmental stress and enhanced cell dying can delay normal cell migration in the formation of the three germ layers, especially the ectoderm, for further brain development. Furthermore, Bad defects involved in three-germ-layers development at 8 hpf were identified by in situ hybridization approach on *cyp26*, *rtla*, and *Sox17* pattern expression markers. Finally, the Bad knockdown-induced severely defected brain was examined by tissue section from 24 to 48 h postfertilization (hpf), which correlated to induce dramatic malformation in the hindbrain. Our data suggest that the BH3-only molecule Bad regulates brain development via controlling programmed cell death on overcoming environmental stress for reducing secondary cell death signaling, which suggests that correlates to brain developmental and neurological disorders in this model system.

## 1. Introduction

Observations of programmed cell death (PCD) in certain model organisms, such as *Caenorhabditis elegans*, *Drosophila melanogaster*, and the mouse (*Mus musculus*), have shaped our understanding of how cells undergo PCD [1,2,3,4,5,6,7]. Various models to explain why cells need to die during development propose reasons for this effect, including the sculpting and deletion of structures, the nutrient supply, the regulation of cell number, and the elimination of abnormal cells [8,9,10]. In many situations, apoptosis inhibition causes embryonic lethality, developmental abnormalities, and various pathologies [1]. Originally, PCD was almost synonymous with apoptosis. More recently, PCD has been classified into apoptosis (Type I cell death), necroptosis (Type II), and autophagy-mediated cell death (Type III)—all pathways that require tight regulation [1,11].

The BCL-2 family of proteins constitutes a critical apoptotic control point residing immediately upstream of the mitochondria. The antiapoptotic members display sequence conservation throughout all four BCL-2 homology domains (BH1–BH4) [11,12]. BH3-only molecules, including BAD, BID, NOXA, PUMA, BIK, and BIM, operate upstream, connecting proximal death signals to the activation of BAX, which permeabilizes the outer mitochondrial membrane to release cytochrome *c* [13]. BAD was the first BH3-only molecule to be connected to proximal signal transduction through its differential phosphorylation in response to extracellular survival factors [14,15]. Dephosphorylated BAD appears to be active and bound to BCL-2 and BCL-XL at the mitochondria; however, when phosphorylated at serine sites (Ser-112, -136, and -155), BAD is inactive and can be bound by 14-3-3 [15,16]. Factors including interleukin-3 (IL-3), insulin-like growth factor 1 (IGF-1), and nerve growth factor transduce intracellular survival signaling by activating kinase cascades that phosphorylate death substrates, including BAD. The phosphatidylinositol 3-kinase (PI3K) pathway, including but likely not restricted to AKT and p70S6K, mitochondrial tethered protein kinase A (PKA), and RSK have been implicated in BAD phosphorylation [17,18,19,20,21,22]. In a loss-of-function approach, proapoptotic BAD suppresses tumorigenesis in the lymphocyte lineage [23]. BAD overexpression in zebrafish induces apoptosis both in vitro and in vivo, which may have biological implications for apoptosis during zebrafish development [24], but very few studies address Bad function in early embryonic development on brain or neurological disorders.

In a zebrafish system, we identified a new role for the BH3-only domain Bad in triggering PCD during early embryonic development, related to completion of the development of tissues or organs, such as the brain, by affecting initial cell migration and the early formation of the three germ layers. These functions and the relationship to environmental stress, cell death signaling, and brain development should all be further addressed.

## 2. Results

### 2.1. The Bad Gene Is a Material Factor Involved in PCD at an Early Developmental Stage

**Figure 1:**
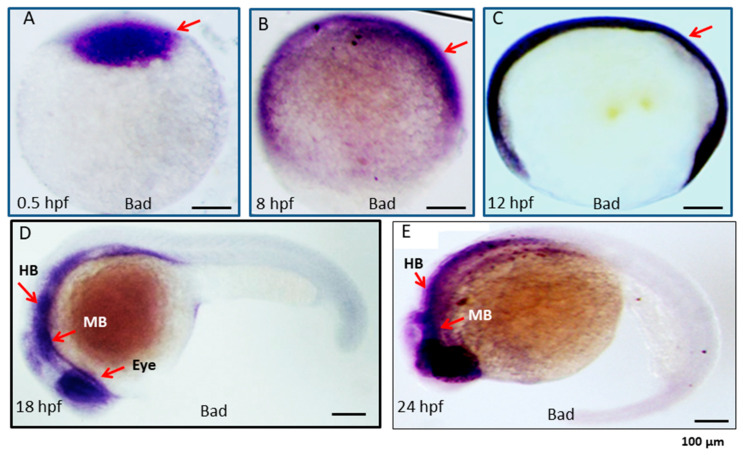
Expression pattern of the proapoptotic gene Bad during embryonic development in zebrafish. Bad expression from early to late developmental stages, as detected by in situ antisense RNA hybridization. *Bad* was visualized by blue staining. Lateral views of embryos are shown in panels (**A**–**E**). (**A**) One-cell stage (half-hour). *Bad* is expressed in all cells examined. (**B**,**C**) At 8 and 12 hpf, *Bad* is expressed throughout the embryo. (**D**) At 18 hpf, *Bad* is expressed and concentrated within the midbrain (MB) and hindbrain (HB) regions (indicated by an arrow) and the eye region. (**E**) At 24 hpf, *Bad* is expressed in the midbrain (MB) and hindbrain (HB) regions (indicated by arrows). Scale bars = 100 µm.

To identify the function of Bad during embryonic development, we first examined the Bad gene expression profile during different developmental stages via in situ hybridization. The Bad mRNA results are analyzed in Figure 1. Bad acts as a material factor at the one-cell stage of the embryo and is then expressed throughout the entire embryo at 8 and 12 h postfertilization (hpf). Moreover, this gene showed dramatically higher specific expression in the ectoderm layer, such as in the middle brain (MB), hindbrain (HB), and the eyes, at 18 and 24 hpf.

**Figure 2:**
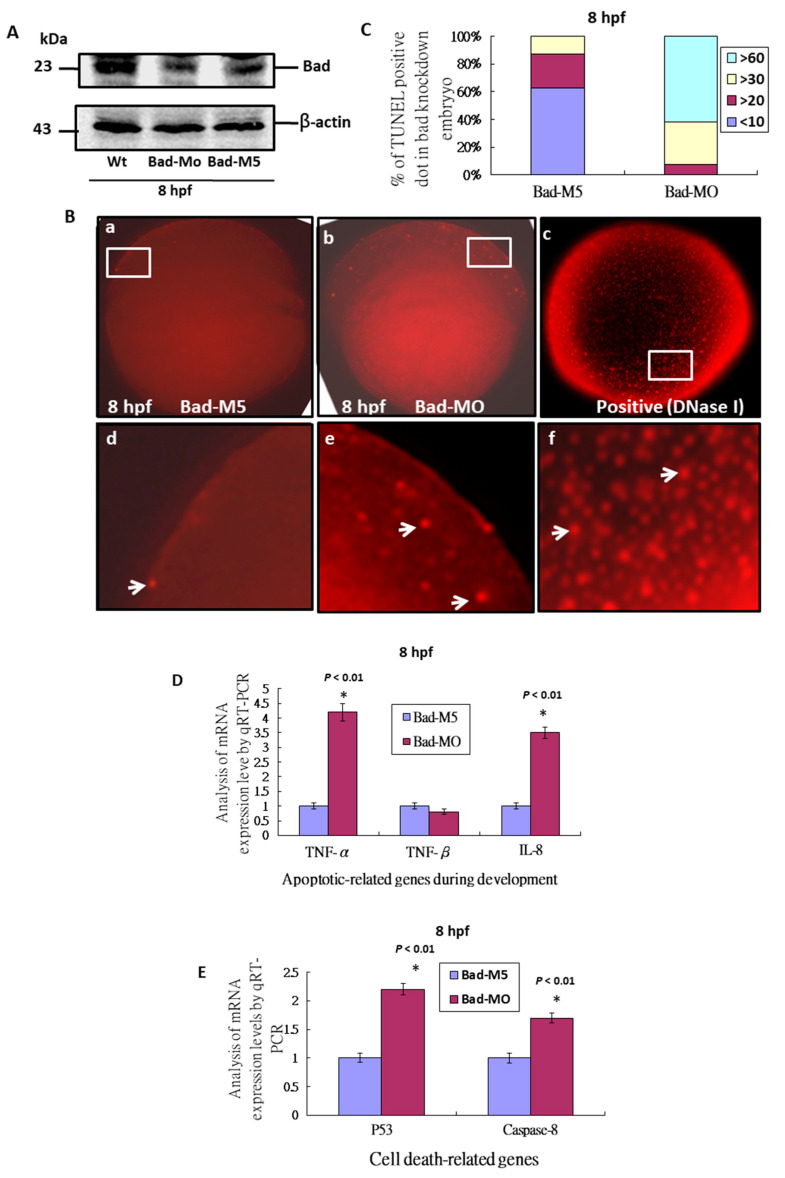
Bad knockdown can enhance p53/caspase-8 death signaling. (**A**) Identification of Bad knockdown by specific morpholino (Bad-MO) at 8 hpf via Western blot analysis. (**B**,**C**) TUNEL-positive spot staining (indicated by arrows) at 8 hpf with Bad knockdown by Bad-MO. The Bad-MO group shows more signal than the Bad-M5 (Control group). TUNEL-positive spots within the embryo were counted and are shown in (**C**). The DNase I treatment group was used as a positive control. (**D**,**E**) Analysis of apoptotic-related genes during development and cell-death-related genes by qRT-PCR at 8 hpf with Bad knockdown. All data were analyzed using either paired or unpaired Student’s *t*-tests, as appropriate. TUNEL-positive cells under the fluorescence microscope are considered apoptotic.

To define Bad gene function during the early embryonic stage, we adopted a knockdown approach. We found that Bad morpholino oligonucleotides (MOs) can effectively knock down Bad expression at 8 hpf (Figure 2A) compared to wild type and the Bad-M5 control. At 8 hpf, Bad knockdown in embryos resulted in extra apoptotic signaling throughout the entire embryo (Figure 2(Bb,Be); indicated by arrows) compared to the Bad-M5 control (Figure 2(Ba,Bd)). The TUNEL-positive spots were counted and are shown in Figure 2C. Up to 65% of embryos in the Bad-Mo group contained 60% TUNEL-positive spots, but the Bad-M5 group showed much smaller numbers, a difference that was statistically significant. Furthermore, we found that delayed PCD induced apoptosis-related genes during development, such as TNF-α and IL-8 (Figure 2D) and the well-known death genes p53 and caspase-8 (Figure 2E). QRT-PCR analysis at 8 hpf revealed significant differences in expression.

### 2.2. The Bad Gene Involved in Regulating Environmental Stress at an Early Developmental Stage

**Figure 3:**
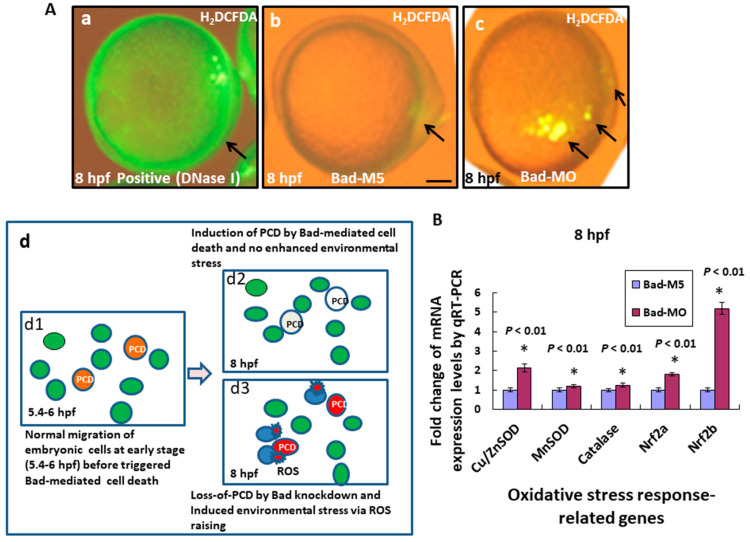
Bad knockdown can enhance ROS production and induces oxidative stress at early embryonic stage. (**A**) Loss-of-Bad-mediated cell death can enhance ROS production. ROS production was assayed using H_2_DCFDA in embryos with Bad knockdown at 8 hpf. The green fluorescent signal, reflecting ROS, is indicated by arrows. Loss of PCD can enhance environmental stress in blue cells; (**A**d3) due to ROS production. Bars indicate 100 μm. (**B**) Analysis of oxidative stress response-related genes by qRT-PCR at 8 hpf with Bad knockdown. All data were analyzed using either paired or unpaired Student’s *t*-tests, as appropriate.

Moreover, Bad knockdown induced severe apoptosis in the embryo. We detected the environmental stress via reactive oxygen species (ROS) production using H_2_DCFDA staining under phase-contrast imaging and found that ROS generation increased by 80% (N = 24) (Figure 3Ac; indicated by arrows) in embryos at 8 hpf compared to the Bad-M5 control (2%) (N = 28) (Figure 3Ab) and the positive control (DNase I treatment; Figure 3Aa). We hypothesize that Bad is required for the complete PCD process, reducing environmental stress during early embryonic development at the start of PCD (5.4–6 hpf; Figure 3Ad1). In its absence, upon entering the later stage (8 hpf; Figure 3Ad3), the living cells (ordinarily Bad-committed death cells) interfered with normal embryonic cell migration and enhanced environmental stress compared to the Bad-mediated committed cell death group (8 hpf; Figure 3Ad2). Increasing environmental stress also upregulated either oxidative stress genes, including Cu/ZnSOD, MnSOD, Catalase, Nrf2a, and Nrf2b (Figure 3B) during development. QRT-PCR analysis at 8 hpf revealed significant differences in expression.

### 2.3. Loss-of-Bad-Mediated PCD Can Interrupt Cell Migration and the Formation of the Three Germ Layers, Especially the Ectoderm

During the gastrula period (5.25–10 hpf) through the end of epiboly, the morphogenetic cell movement of involution, convergence, and extension occurs. The three germ layers, the ectoderm (derived from the epiblast), the mesoderm, and the endoderm (derived from the hypoblast), also develop during this stage [25].

**Figure 4:**
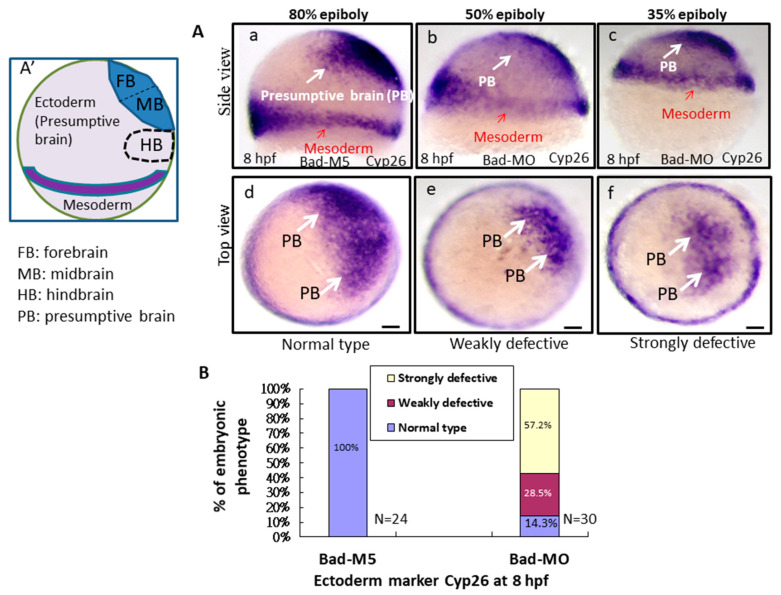

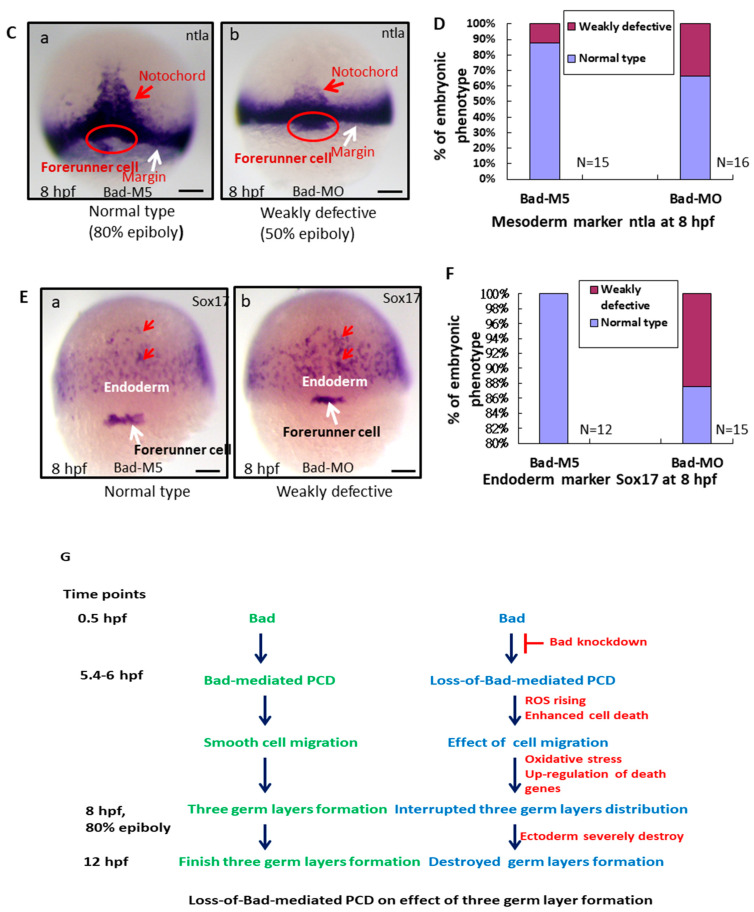
Bad-mediated PCD can regulate cell movement and cells in three-germ-layer targeting to destination sites, as shown via in situ hybridization. Morphological analysis of embryos injected with either 25 ng of control-MO (Bad-M5) or Bad-MO and examined at 8 hpf after ectoderm, mesoderm, and endoderm tissues were stained with *cyp26*, *ntla*, and *sox17*, respectively, via in situ hybridization. (**A**) The embryos are stained with *cyp26* (side view, panels a–c; top view, panels d–f), with the presumptive brain (PB) indicated by white arrows and the mesoderm by red arrows. (**B**) Quantification of loss-of-Bad-induced PCD effects on ectoderm migration in connection to abnormal brain development at 24 hpf. All data were analyzed using either paired or unpaired Student’s *t*-tests, as appropriate. *p* < 0.01. (**C**) Bad knockdown can induce mild mesoderm defects, as shown by probing with the mesoderm marker ntla gene. Panels a (Bad-M5 group) and b (Bad-MO group): notochord profile indicated by red arrows; margin pattern indicated by white arrow; and forerunner cell indicated by red circle. Bars indicate 100 μm. (**D**) Quantification of mild loss-of-Bad-induced PCD effects on mesoderm pattern. All data were analyzed using either paired or unpaired Student’s *t*-tests, as appropriate. *p* < 0.01. (**E**) Bad knockdown can induce very mild endoderm defects, as shown by probing with the endoderm marker sox17 gene. Panels a,b, endoderm signal indicated by red arrows and forerunner cell by white arrow. The defect ratio was counted and is shown in (**F**). All data were analyzed using either paired or unpaired Student’s *t*-tests as appropriate. *p* < 0.01. (**G**) Sketch illustrating how loss-of-Bad-mediated PCD affects smooth cell migration during the formation of the three germ layers at 8 hpf: environmental stress in the form of ROS production was increased, and enhanced cell death further interrupted brain development at a later developmental stage.

To better determine the effect of Bad-mediated cell death on reducing environmental stress, the early embryonic cell migration and development of the three germ layers were characterized. At 8 hpf, while detecting the ectoderm distribution in the presumptive brain using a *Cyp26* marker probe, we found that Bad knockdown severely affected the ectoderm distribution, resulting in embryos defined as weakly defective (50% epiboly status) (Figure 4Ab,e) and strongly defective (35% epiboly status) (Figure 4Ac,f) compared with the Bad-M5 group used as a normal control group (80% epiboly status) (Figure 4Aa,d). The percentages of defective embryos are shown in Figure 4B, reflecting a significant difference. Then, by tracing the mesoderm distribution with the marker *ntla* gene, with 80% epiboly status, we found that Bad knockdown exerted a partial effect on the mesoderm distribution notochord (Figure 4Cb), reaching 50% epiboly status compared with the Bad-M5 group (Figure 4Ca). The notochord-delayed development ratio exhibited up to a 20% difference, as shown in Figure 4D. Interestingly, upon tracing the endoderm with *Sox17*, we found only a mild difference in the endoderm distribution (Figure 4E), which exhibited approximately 12% weakly defective embryos compared to the Bad-M5 group, as shown in Figure 4F. In summary, Bad defects can induce abnormal distribution of the three germ layers through enhanced environmental stress with increasing ROS and cell apoptosis (Figure 4G).

### 2.4. Bad-Mediated PCD Can Regulate Brain Morphogenesis

**Figure 5:**
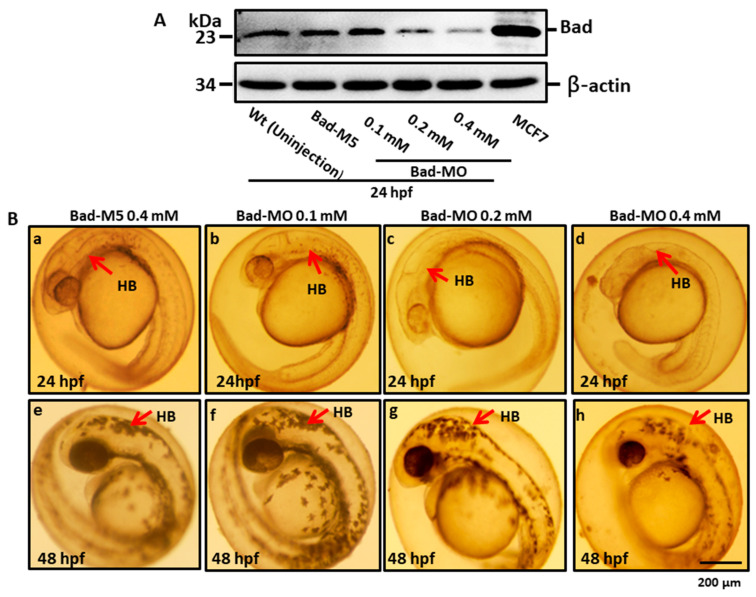

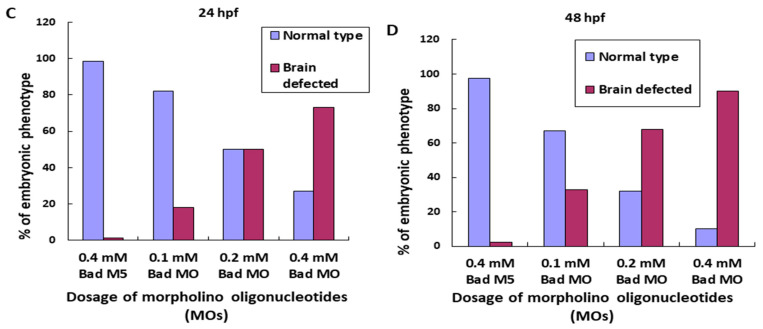
BH3-only proapoptotic gene Bad can regulate brain development between 24 and 48 hpf. (**A**) Dosage dependence of Bad knockdown (lanes 3–5 for 0.1, 0.2, and 0.4 mM, respectively) shown by Western blot analysis and compared with the uninjected group (lane 1) and the normal control Bad-M5 group (lane 2). The positive control MCF7 cell lysate is shown in lane 6. (**B**–**D**) Identification of morphological phenotype defects in the brain during Bad knockdown with dosage response. Each one-cell-stage embryo was injected with either 0.4 mM of control-MO or 0.1, 0.2, or 0.4 mM of Bad-MO. At 24 and 48 hpf, embryos were observed under a microscope. Phase-contrast images of the control-MO-injected (**B**a,e) and Bad-MO [lower dose (0.1 mM)-injected; (**B**b,f)] embryos show normal brain development. However, the embryos injected with higher doses of Bad-MO embryos show abnormal brain development ((**B**c,g), 0.2 mM; (**B**d,h) 0.4 mM at 24 and 48 hpf, respectively) in the hindbrain (HB), indicated by arrows. The defect rates (*N*, up to 120 embryos) were counted and are shown in Figure 3C,D for 24 and 48 hpf, respectively, revealing a statistically significant difference.

We further determined the effects of reducing Bad expression later than the strong effects on ectoderm migration at 8 hpf on brain morphogenesis. A dosage-dependent screening approach was applied at 24 hpf. Using Western blot, we determined that morpholino-mediated Bad knockdown showed a dosage-dependent response (Figure 5A, lanes 3–5) compared with the Bad-M5 (Figure 5A, lane 2) and uninjected groups (Figure 5A, lane 1), with significant differences. Next, we traced the brain development by stereomicroscopy at 24 and 48 hpf under Bad knockdown. The brain developmental defects are shown in Figure 5B, which reveals shortened and widened brains (Figure 5(Bg,Bh) for 0.2 and 0.4 mM; indicated by arrows) at 48 hpf compared with the Bad-M5 0.4 mM and Bad-MO 0.1 mM groups. The instances of this phenotype were counted, and the results are shown in Figure 5C (at 24 hpf) and Figure 5D (at 48 hpf): namely, a gradual dosage-dependent increase in the brain defect rate, up to 60% and 80% at 24 and 48 hpf, respectively, in the 0.4 mM group.

### 2.5. Dramatic Malformation Was Observed in the Hindbrain during Knockdown of BAD

**Figure 6:**
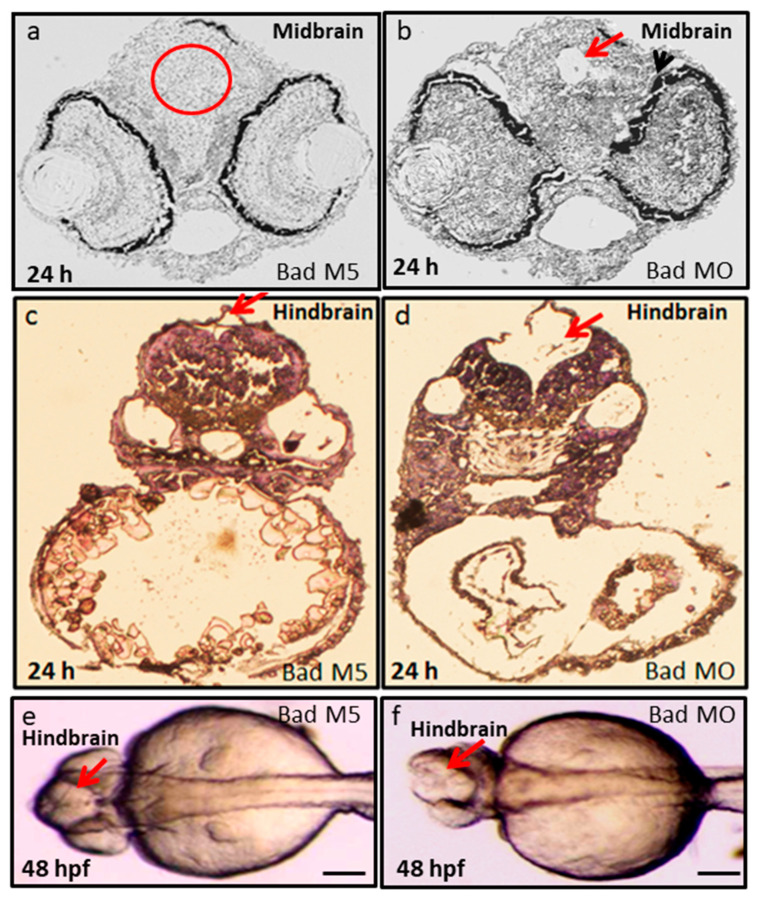
Bad loss can produce brain defects and brain malfunction between 24 and 48 hpf. Dissection and observation of brain development in loss-of-Bad-mediated PCD embryos at 24 and 48 hpf. HE staining to show midbrain and hindbrain development in the Bad-M5 group in panels **a**–**c**; and the Bad-Mo group in panels (**b**,**d**), at 24 and 48 hpf, respectively, as indicated by red arrows and red circle. Phase-contrast image for observation of brain morphology in (**e**) (Bad-M5) and (**f**) (Bad-MO) at 48 hpf. Brain malfunction is indicated by arrows. Scale = 200 μM.

Furthermore, tissue dissection and hematoxylin and eosin (HE) staining were performed to observe the midbrain and hindbrain morphogenesis. We found that Bad knockdown induced midbrain development defects, as shown in Figure 6b (indicated by arrow), compared to Bad-M5 (Figure 6a). Dramatic malformation was observed in the hindbrain, as shown in the dissection image (Figure 6d; indicated by arrow) and the Nomaski image (Figure 6f; indicated by arrow) at 48 hpf compared to the Bad-M5 groups (Figure 6c,e, respectively).

### 2.6. Investigation of the Correlation of Bad-Mediated Cell Death with Brain Function through the Swimming Ability Test

**Figure 7:**
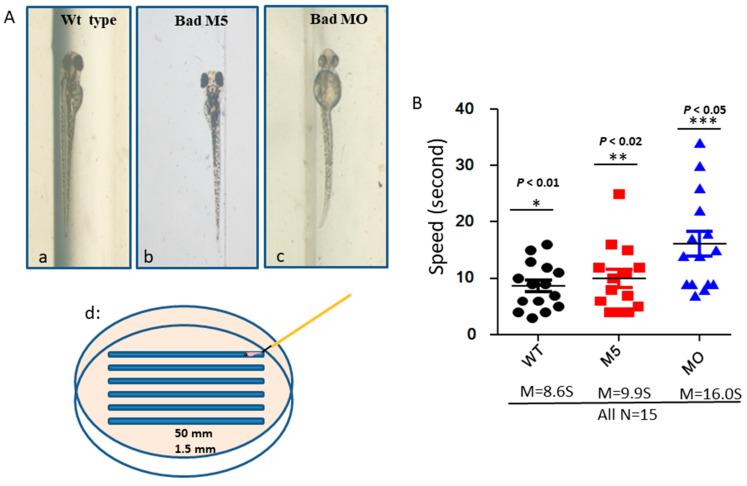
Identification of brain functions through monitoring of swimming behavior at 72 hpf. (**A**) Bad knockdown fish lines at 72 hpf. The phenotypes of the wild-type group show mild brain defects (**A**b). The 50 mm × 1.5 mm slot for swimming is displayed in (**A**d). (**B**) Swimming speed over a limited length was monitored for different fish lines with and without Bad knockdown. The wild-type with Bad-MO group was slower than the other groups by approximately 5 s at 72 hpf. All data were analyzed using either paired or unpaired Student’s *t*-tests as appropriate.

To determine whether Bad defects can regulate brain function based on swimming ability, we designed a swimming ability test using a slot in agarose gel (1.5 mm × 50 nm), as shown in Figure 7Ad. This test was completed in limited time periods by fishes under different conditions (Figure 7(Aa–Ac)). After testing (Figure 7B), we found that the fastest group was the wild-type uninjected group (8.6S), while the second fastest group was the wild-type with Bad-M5 group (9.9S). The final group was the wild-type with Bad-MO group (16.0S), which exhibited up to 6-s differences in swimming ability compared to the other groups, further demonstrating that ROS/p53/caspase-8 death signaling is involved in brain development and brain function, based on these statistically significant differences.

## 3. Discussion

In the zebrafish model system, Bad is first reported as a material factor with expression during the very early embryonic stage, from the one-cell stage to the brain developmental stage at 24 hpf. Upon loss-of-function induced by MOs, the embryos exhibited increased cell death via a TUNEL assay and signs of induced oxidative stress. These conditions also delayed cell migration and further interfered with the development of the three germ layers. Interestingly, this blockage of cell migration affected ectoderm development, especially in terms of brain morphogenesis and malfunction. Thus, Bad-mediated PCD is required to block either environmental stress genes or the upregulation of the death genes p53 and caspase-8 between the gastrula stage (8 hpf) and 24 hpf, which reduced the swimming function during Bad knockdown.

### 3.1. Bad Loss-of-Function Can Enhance Cell Death Signaling

The recently emerged topic of PCD is taking on new significance. Starting in the 2000s, many studies have focused on the function of the Bcl-2 family genes during embryonic development, especially on BH3-only domain members such as Bax, Bak, and Bad in the higher vertebrate mouse system, which possesses sequence homology only within the amphipathic α-helical segment that constitutes the critical death domain [14,26]. Doubly deficient cells revealed that the BAX and BAK molecules constitute requisite gateways to the mitochondria [27] and the endoplasmic reticulum [28] to control apoptosis. *Bid-*deficient mice are resistant to pathological Fas-activated hepatic apoptosis [29]. *Bim-*deficient mice are defective in eliminating reactive thymocytes [30].

However, early neural development is not impaired in mice deficient in Bad or mutant mice homozygous for a mutant form of Bad that cannot be phosphorylated in response to survival factors [31]. These results suggest that Bad phosphorylation is not essential for regulating survival within early neural precursors.

After a decade, we revisited this issue using the zebrafish system, which has many advantages, such as producing many embryos in one mating and greater transparency. In this system, we found that Bad as a material factor can express in one cell stage, which then expressed in whole embryo at 12 hpf. Final, Bad expressed in middle and hindbrain area at 18 and 24 hpf, which was indeed required for early embryonic development (Figure 1) and complete tissue and organ morphogenesis (Figure 2, Figure 4 and Figure 5). It is very interesting finding during knockdown of Bad can enhance secondary death signaling wave such as TNF-α and IL-8 (Figure 2D) and p53 and caspase-8 (Figure 2E) as a new concept in molecular embryonic development.

### 3.2. Reactive Oxygen Species (ROS) Act as Environmental Stress Factors That Regulate Cell Death during Early Embryonic Development

Redox status influences multiple cellular processes, including cell proliferation, differentiation, and signaling. Through these events, ROS play a significant role in embryonic development. In many examples, because ROS production is essential for mediating apoptosis and cell elimination [32], oxidative stress and redox-related signaling are vital to normal developmental processes, such as progression to the blastocyst stage, neuronal differentiation, and digit formation. In those dynamic events, the inability to increase oxidative stress leads to abnormal developmental sequences and dysregulation. Nonetheless, excess oxidative stress is strongly associated with embryotoxicity. Normal and abnormal ROS levels depend on antioxidant systems in the embryo, which modulate developmental redox status with physiological and phenotypic consequences [33,34].

In our system, we found that Bad knockdown can enhance ROS generation (Figure 3A) and is correlated with the induction of oxidative stress, which also induced some stress-response genes, especially Cu/ZnSOD, Nrf2a, and Nrf2b (Figure 3B), under further enhanced environmental stress (Figure 3Ad1–3) [33,34]. Correlations were also identified with the induced expression of apoptotic genes, such as TNF-α, p53, and caspase-8, and eventually the apoptosis-induced gene IL-8 [1].

### 3.3. Loss of Bad-Mediated PCD Can Interfere with Ectoderm Development, Especially Brain Development, via Delayed Early Cell Migration

Based on early studies, in the early stage, PCD must be triggered to remove unwanted cells that regulate the normal cell for smooth migration during epiboly, which also requires an engulfing dying cell system, such as one involving phosphatidylserine receptors [35]. Mutant mice deficient in PSR exhibit expanded neural tissues [36], and knockdown of PSR exhibited accumulation of apoptotic cells throughout the embryo and induced brain and heart defects in zebrafish [36,37]. In this study, we found that the Bad-loss-mediated enhanced apoptosis process can interfere with cell migration and disrupt germ layer formation, especially in the ectoderm (Figure 4A), which is required for brain development. We have proposed a new concept that early PCD is required for smooth-cell migration and germ layer formation, especially affecting ectoderm layer migration (Figure 4A), which exhibits a delay of approximately 30–45% in epiboly at this stage during Bad knockdown. Cell death is also required for the PSR-mediated clearance of the apoptotic cell system. Then, it starts at approximately 12 hpf. PCD is detected within the neural cells of zebrafish embryos at 12 hpf after the neuroectoderm becomes morphologically distinct [38,39]. Then, PCD is localized within the rostral half of the developing central nervous system (CNS), extending caudally as development proceeds [38,39]. PCD affects neuronal precursors or newly differentiated neurons before the onset of axon extension, which occurs at 16 hpf [40]. This proposed process is strongly supported by the mouse system: the earliest PCD affecting the presumptive neural tissue is observed within the anterior distal epiblast, which is destined to become the neural plate during gastrulation at E6.5 [41]. Apoptotic cells are also found in the hindbrain neural folds between E8 and E9 (defined as second-round PCD) [42]. Then, between E12 and E16, PCD occurs within the ventricular and intermediate zones of the cerebral cortex, which consist of proliferating precursors and newly postmitotic neuroblasts [43,44,45]. In our system, we found that Bad knockdown also enhanced apoptotic cell death in the whole embryo at 8 hpf (Figure 2B–E), which also required p53/caspase-8 upregulation.

### 3.4. Bad Knockdown-Induced Brain Defect during Development

Traditionally, dying cells were thought to have limited signaling capacity, as they are rapidly cleared by phagocytes. However, it is now clear that apoptotic cells release many signals that profoundly affect their cellular environment. These signals include mitogens as common regulators of neural development and PCD, which promote proliferation and tissue repair, and death factor, which stimulates coordinated cell killing [9].

In the nervous system, p53 plays a role in the elimination of new postmitotic neurons that do not differentiate appropriately. Several studies have shown that p53 is involved in the natural cell death of peripheral neurons of the sympathetic superior cervical ganglion soon after birth [46,47,48].

In our system, we have found that the loss of Bad function led to enhanced ROS generation and triggered more PCD, which correlated with the induction of the p53/caspase-8 death signals [49,50,51] and further activation of Bid to cleaved tBid [52], which can work on mitochondria or regulate downstream caspase-3 activation, which correlated to brain development, as inducing dramatic malformation (Figure 6) and biological function in terms of swimming behavior (Figure 7) [11,53] and may suggest that Bad is involved in mammalian brain and neuropathological disorders.

**Figure 8:**
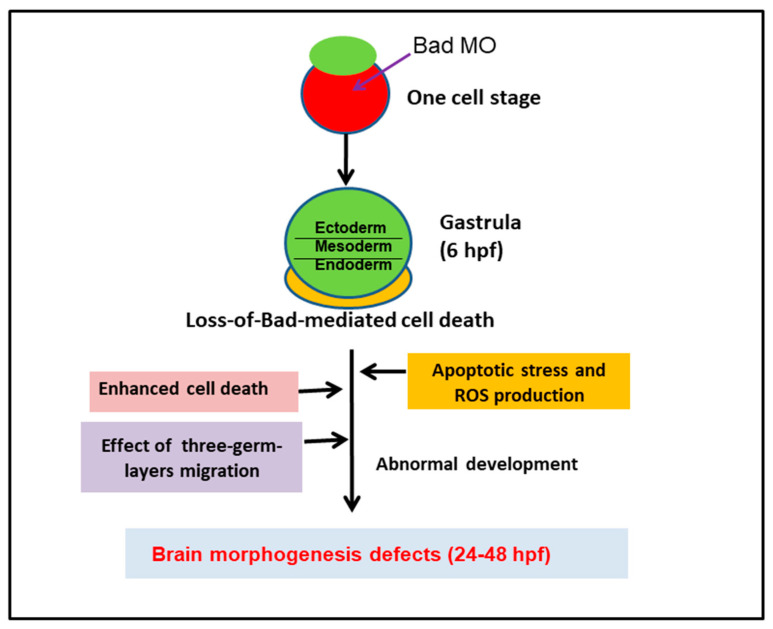
Diagram of Bad involvement in committed PCD and brain development from 0.5 to 72 hpf, including both first- (5.4–12 hpf) and second-round PCD (after 12 hpf). The early PCD is essential for early smooth-cell migration and for the formation of the three germ layers in later development, which is connected to second-round PCD for tissue and organ development.

In summary (Figure 8), the BH3-only proapoptotic Bad is expressed as a material factor at the early developmental stage, i.e., at 0.5 hpf. If knockdown of this death factor occurs at the one-cell stage, then at 8 hpf, the loss of Bad-mediated PCD (PCD begins at 5.4–6 hpf) produces severe environmental stress with heightened ROS production throughout the embryo, which is also correlated with enhanced PCD induction. At 8 hpf, Bad defect enhanced PCD dramatically influenced early embryonic cell migration, targeting specific sites, and destroyed the formation of the three germ layers, especially the ectoderm (epiblast) for brain development. Interestingly, loss-of-Bad-mediated cell death also affected normal brain development at 24 to 48 hpf, which correlated with the triggering of a novel cell death pathway, ROS/p53/caspase-8. The Bad-mediated cell death is required for three-germ-layers migration, which especially correlated to brain development and nervous biological function.

## 4. Materials and Methods

### 4.1. Experimental Fish

The wild-type AB strain was used for MO injection. All fish lines were reared in a circulating system under a 14-h/10-h light/dark photoperiod illumination cycle, maintained under standard conditions at 28.5 °C, and were staged based on hpf, as previously described [40]. Techniques for the care and breeding of zebrafish have been previously described in detail [25].

### 4.2. Apoptotic Cell Staining

The AB strain (wild type) embryos at the one- or two-cell stage were injected with Bad-MO or control-MO (as Bad-M5) using a gas-driven microinjector (Medical System Corporation, Greenvale, NY, USA). The positive control groups were treated with DNase I (0.1 μg/mL) for 4 h. For AO staining, embryos were harvested at 24 hpf and fixed with 4% paraformaldehyde in PBS (pH 7.4) at room temperature for 30 min. The embryos were stained with AO (1 μg/mL) for 3–5 min, washed twice with PBS, and evaluated by fluorescence microscopy (using incident light at 488 nm excitation, with a 515 nm longpass filter for detection) [35]. For the TUNEL assay (in situ cell death detection kit, Roche Diagnostics, Indianapolis, IN, USA), the embryos were also fixed in paraformaldehyde at the end of the incubation period (8 and 24 hpf) and were then dechorionated and incubated in blocking solution (0.1% H_2_O_2_ in methanol) for 30 min at room temperature to block endogenous peroxidases. Embryos were rinsed with PBST, incubated on ice in a solution of 0.1% Triton X-100 in 0.1% sodium citrate for 30 min to increase permeability, and rinsed again twice with PBST. Afterwards, the embryos were incubated with tetramethylrhodamine (TMR)-conjugated nucleotides and terminal deoxynucleotidyl transferase at 37 °C for 1 h. The embryos were analyzed for positive apoptotic cells under a fluorescence microscope equipped with a spot II cool CCD (Diagnostic Instruments, Sterling Heights, MI, USA) [35].

### 4.3. ROS Detection

ROS levels were determined in zebrafish embryos by staining with 10 µM H_2_DCFDA (Invitrogen), a reliable fluorogenic marker of ROS production in zebrafish embryos, at room temperature for 1 h [54]. The samples were examined immediately under a fluorescence microscope equipped with a spot II cool CCD (Diagnostic Instruments, Sterling Heights, MI, USA) to detect the fluorescein.

### 4.4. RNA Extraction

To obtain a sufficient quantity of RNA, 30 embryos were pooled as a sample. Samples were homogenized in 0.6 mL TRIzol Reagent (Invitrogen, Carlsbad, CA, USA). After chloroform extraction, the total RNA samples were purified and treated with DNase I to remove the genomic DNA using an RNeasy Mini Kit (Qiagen, Huntsville, AL, USA). The quantity and quality of total RNA were assessed by A-drop (FastGene) and agarose gel electrophoresis, respectively [35].

### 4.5. Reverse-Transcription Polymerase Chain Reaction (RT-PCR) Analysis

For cDNA synthesis, 5 μg of total RNA was reverse transcribed in a final volume of 20 μL containing 1 mM dNTPs, 5 μM oligo(dT)_18_, 1 unit of an RNase inhibitor, and 10 units of RevertAid M-MuLV reverse transcriptase (Thermo Scientific, Vilnius, Lithuania) for 5 min at 72 °C, followed by incubation for 60 min at 42 °C. For PCR amplification, 1 μL of cDNA was used as a template in a 20 μL final reaction volume containing 0.25 μM dNTP, 1.25 units of Ex Taq DNA polymerase (TaKaRa, Shiga, Japan), and 0.2 μM of each primer [35]. The primer sets are given in Table 1.

### 4.6. Quantitative (q)RT-PCR

The mRNA expression levels were measured by qRT-PCR with a Roche Lightcycler Nano (Roche, Penzberg, Germany). The final volume in a well was 20 μL and contained 4 μL of OmicsGreen qPCR 5X master mix (Omics Bio, Taipei, Taiwan), 3.2 ng of cDNA, and 50 nM of primer pairs. Cycling parameters were as follows: 95 °C × 900 s, followed by 45 cycles of 95 °C × 15 s, 60 °C × 20 s, and 72 °C × 20 s. The standard curve of each gene was checked in the linear range, with β-actin as an internal control [54]. The primer sets are given in Table 2.

### 4.7. Morpholino Oligonucleotides (MOs)

The morpholino-modified antisense oligonucleotide was purchased from Gene Tools (Philomath, OR, USA). The MO used against *bad* begins at (5′-CAGaGATAtTAAAcAtATGTGc**CAT**-3′). The maximal dosage that caused no obvious toxic effects on embryogenesis was used as follows: Bad-MO at 8 ng/embryo and control MO (5′-CAGCGATAATAAAGAAATGTGGCAT-3′) at 8 ng/embryo. The MOs were prepared using Danieau solution (58 mM NaCl, 0.7 mM KCl, 0.4 mM MgSO_4_, 0.6 mM Ca(NO3)_2_, and 5.0 mM HEPES; pH 7.6). The MO solution (8 ng/embryo) containing 0.1% phenol red (a visualizing indicator) was injected into zebrafish embryos at the one-cell stage using a gas-driven microinjector (Medical System Corporation, Greenvale, NY, USA) as previously described. Wild-type embryos without injection were subjected only to Bad protein measurement and morphological observation [35].

### 4.8. Western Blotting

The embryos were injected with *bad* MO and control MO at the one-cell stage using a gas-driven microinjector (Medical System Corporation, Greenvale, NY, USA). Fifty embryos were collected at 24 hpf and homogenized in lysis buffer (10 mM Tris-HCl, pH 6.8, 20% glycerol, 10 mM sodium dodecyl sulfate (SDS), and 2% β-mercaptoethanol). An aliquot of each lysate with 40 μg protein per sample was separated by electrophoresis on an SDS polyacrylamide gel to resolve the proteins. The gels were immunoblotted with the following antibodies: anti-bad antibodies (BD Transduction Laboratories) and anti-β-actin monoclonal antibodies (BD Pharmingen), followed by peroxidase-labeled goat anti-mouse secondary antibodies (1:15,000 dilution; Amersham Biosciences, Piscataway, NJ, USA) or peroxidase-labeled goat antirabbit secondary antibodies (1:7500 dilution; Amersham Biosciences, Piscataway, NJ, USA). Chemiluminescence indicative of antibody binding was captured by MultiGel-21 (TOP BIO, Taipei, Taiwan) as previously described [55].

### 4.9. Whole-Mount In Situ Hybridization

Fragments of *Bad, cyp26a1* [56], *ntl* [57], and *sox17* [58] were obtained by PCR and inserted into the pGEM-T easy vector (Promega, Madison, WI, USA). The inserted fragments were amplified by PCR using T7 and SP6 primers, and the products were used as templates for in vitro transcription with T7 and SP6 RNA polymerases (Roche) in the presence of digoxigenin (DIG)-UTP (Roche) to synthesize sense and antisense probes. Zebrafish embryos were anesthetized on ice and fixed with 4% paraformaldehyde in a phosphate-buffered saline (PBS; 1.4 mM NaCl, 0.2 mM KCl, 0.1 mM Na_2_HPO_4_, and 0.2 mM KH_2_PO_4_; pH 7.4) solution at 4 °C overnight. Afterwards, the samples were washed several times for 10 min each with diethylpyrocarbonate (DEPC)-treated PBST (PBS with 0.1% Tween-20). After PBST washing, the samples were incubated with hybridization buffer (HyB, 50% formamide, 5X SSC, and 0.1% Tween 20) at 65 °C for 5 min and with HyB containing 500 μg/mL yeast tRNA at 65 °C for 2 h before hybridization. After overnight hybridization with 0.5 μg/mL DIG-labeled antisense or sense RNA probes, the embryos were serially washed with 50% HyB in 2X SSC (at 65 °C for 20 min), 2X SSC (at 65 °C for 10 min), 0.2X SSC (at 65 °C for 30 min, twice), and PBST (at room temperature for 10 min). Afterward, the embryos were immune-reacted with an alkaline phosphatase-coupled anti-DIG antibody (1:8000) and stained with nitro blue tetrazolium (NBT) (Roche) and 5-bromo-4-chloro-3-indolyl phosphate (BCIP) (Roche) for the alkaline phosphatase reaction. The in situ hybridization assay used embryos injected with *bad***-**MO and control-MO at 8, 24 and 72 hpf [35,37].

### 4.10. Swimming Activity Assay

To determine the swimming activity of zebrafish larvae at 3 day postfertilization (dpf), the larvae were tested in slots in an agarose plate (50 mm × 15 mm; length × width) in three different groups at 28.5 °C. Individual fish were tested in five repeats by stimulation with a brush.

## Figures and Tables

**Table 1:** Primer sequences was used gene cloning.

Gene	Forward 5′→3′	Reverse 5′→3′
*p53*	CTACTAAACTACATGTGCAATAGCAG	CTGAGGCAGGCACCACATCACT
*bad*	ATGGCACATATGTTTAATATCTCTGA	CTACTCTGCGGGGCGCGA
*fabp7a*	CTCTCAACATGGTCGATGCATT	CTGGACATTATGCCTTCTCGTA
*pax2a*	CTTCTAACAGGCACATCCCAT	CATTAACCCTCACTAAAGGGAACTATCCGTTCAAAGCCCG

**Table 2:** Primer sequences was used for real-time quantitative PCR.

Gene	Forward 5′→3′	Reverse 5′→3′
*β* *-actin*	ACTGTATTGTCTGGTGGTAC	TACTCCTGCTTGCTAATCC
*p53*	ACCACTGGGACCAAACGTAG	CAG AGTCGCTTCTTCCTTCG
*Caspase-8*	CCAGACAATCTGGATGAACTTTAC	TGCAAACTGCTTTATCTCATCT
*pva1b5*	ATGGCACTTGCAGGAATCCTGA	TGTTGGTCTCGGCCTCTGTGAG
*crybb1*	ATGTCTCAGACCGCCAAATCCG	GCCCTGGAAGTTCTCCTGGTCA
*pax7a*	CCAGGAACAGTTCCTCGAATGATG	CCGTGATGGGCCATTTCCAC
*irx4a*	GCGGACAAGGCTACGGGAATT	AGCGTTTTCCTGCGGGTCC
*fabp7a*	TGTGCCACTTGGAAACTGGTTGAC	CCCAGTTTGAAGGAGATCTCGGTG
*Catalase*	TAAAGGAGCAGGAGCGTTTGGCTA	TTCACTGCGAAACCACGAGGATCT
*Mn–sod*	CCGGACTATGTTAAGGCCATCT	ACACTCGGTTGCTCTCTTTTCTCT
*Cu/Zn–sod*	GTCGTCTGGCTTGTGGAGTG	TGTCAGCGGGCTAGTGCTT
*nrf2a*	GAGCGGGAGAAATCACACAGAATG	CAGGAGCTGCATGCACTCATCG
*nrf2b*	GGCAGAGGGAGGAGGAGACCAT	AAACAGCAGGGCAGACAACAAGG
